# Low-grade ductal carcinoma in situ (DCIS) arising in a fibroadenoma of the breast during 5 years follow-up: A case report

**DOI:** 10.1097/MD.0000000000024023

**Published:** 2021-03-12

**Authors:** Hiroko Shojaku, Ryota Hori, Toru Yoshida, Kazuhiro Matsui, Katsuo Shimada, Nobutatsu Takayanagi, Kyo Noguchi

**Affiliations:** a Department of Radiology, University of Toyama; b Department of Radiology; c Department of Surgery; d Department of Pathology, Saiseikai Takaoka Hospital; e Department of Surgery, Imizu Municipal Hospital, Toyama; f Department of Pathology, Medical Center of Toyama City Medical Association Toyama, Japan.

**Keywords:** DCIS, dynamic CT, dynamic MRI, FA, US

## Abstract

**Rationale:**

Fibroadenoma (FA) is a common type of benign breast tumors but ductal carcinoma in situ (DCIS) rarely arises within this tumor type.

**Patient concerns:**

This case report presents a non-symptomatic 61-year-old woman with FA that was coincidentally found during a breast cancer screening program performed 5 years ago by her city of residence. She had subsequently been followed-up with mammography and breast ultrasound (US). US showed a slightly enlarged tumor and dynamic magnetic resonance imaging (MRI) indicated malignancy within the FA.

**Diagnosis:**

The pathological examination revealed low-grade DCIS within the FA.

**Interventions:**

The patient underwent a core needle biopsy followed by breast-conserving therapy with sentinel lymph node biopsy and then postoperative radiation therapy.

**Outcomes:**

Currently, she has been followed-up for 2 years without no signs of recurrence.

**Lessons:**

Careful observation with US followed by dynamic MRI is essential in the early diagnosis of DCIS originating in a FA.

## 1 Introduction

Fibroadenomas (FAs) are the most common type of benign tumor that often present in women both before and after menopause. They gradually decreased in size in parallel with the decrease effects of estrogen, especially after the late 40s, and sometimes evolve to degenerative fibroadenoma (FA). The incidence of carcinoma arising from a FA is infrequent with a reported range of only 0.002% to 0.1%.^[1,2]^

Here, we highlight a case of low-grade ductal carcinoma in situ (DCIS) breast cancer that arose in a FA and was followed-up over 5 years by 2 hospitals. Careful observation with the ultrasound (US) followed by dynamic magnetic resonance imaging (MRI) was essential in the early diagnosis of the DCIS originating in the FA in this case. The excised tumor encapsulated with thin fibrous tissue was composed of an intense proliferation of ductal cells showing cribriform, tubular, and solid growth patterns with fibroadenomatous myxoid stroma.

## 2 Case presentation

**Figure 1:**
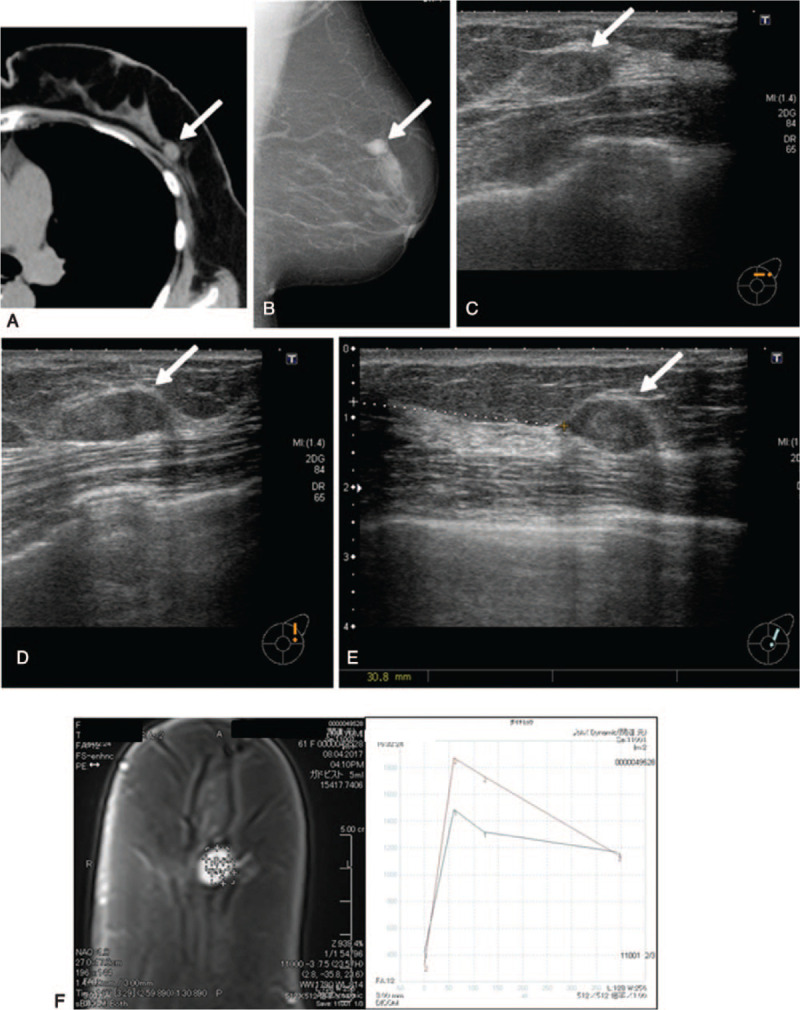
Imaging findings. (a) Chest CT shows a 12-mm ovoid homogeneous mass with defined margin in the left breast C area (arrow). (b) Mammography shows a 16-mm high-density tumor without calcification (arrow). (c–e) US reveals a thick, disk-shaped, slightly inhomogeneous low-echogenic tumor (arrow) with a defined margin with lateral echo, gained echo, and no halo sign. The tumor measures 15.8 mm × 14.1 mm × 7.5 mm and is slightly larger especially in thickness than that measured 5 years ago. The nipple-to-tumor distance was 31 mm in the 1 o’clock direction. (f) The time–intensity curve of dynamic MRI shows a malignant pattern with rapid enhancement and washout.

A 61-year-old non-symptomatic Japanese housewife presented to our hospital because of an abnormality on chest radiography undergone during a lung cancer screening program in her city of residence. She had no family history of breast cancer and was taking no medications. She had a painless 1.5-cm elastic hard palpable mass in her left breast C area. There were no abnormalities in the clinical laboratory examination. Chest computed tomography (CT) showed old left pulmonary inflammation, and also revealed an ovoid homogenous mass with regular margin measuring 12 mm in her left breast C area (Fig. 1a). Mammography showed a well-defined high-density ovoid-shaped mass without calcification that measured 14 mm × 11 mm in the M area on the mediolateral oblique view (Fig. 1b) and measured 16 mm × 11 mm in the O area on the craniocaudal view. US showed a thick, disk-shaped, slightly inhomogeneous low-echogenic tumor (Fig. 1c–e). The tumor had a defined margin with lateral echo. The tumor size was 15.8 mm × 14.1 mm × 7.5 mm.

Five years before this consultation, she had undergone a mammographic examination following a breast cancer screening program performed by her city residence that incidentally revealed a density mass in her of left breast that measured 16 mm × 12 mm in the M area on the mediolateral view and 15 mm × 14 mm in the O area on the craniocaudal view (Table 1). She then underwent a breast cancer check-up at our hospital. Ultrasound showed a tumor size of 14.6 mm × 14.2 mm × 6.6 mm. It was diagnosed as class II on fine needle aspiration biopsy.

**Table 1:** Clinical course.

(1) Five years before the diagnosis of ductal cancer in situ (DCIS) in FA
Her left breast mass was found by mammography incidentally, and fine needle aspiration biopsy by ultrasound (US) showed a class II.
(2) Three years before the diagnosis of ductal cancer in situ (DCIS) in FA
The dynamic CT showed the benign pattern and the core needle biopsy showed FA.
(3) At the diagnosis of ductal cancer in situ (DCIS) in FA
Because of slightly enlargement of her tumor by US, the dynamic magnetic resonance imaging (MRI) was undertaken which showed a malignant pattern. The histology of her resected breast tumor indicated the ductal carcinoma in situ within FA.

CT = computed tomography, DCIS = ductal carcinoma in situ, FA = fibroadenoma, US = ultrasound.

**Figure 2:**
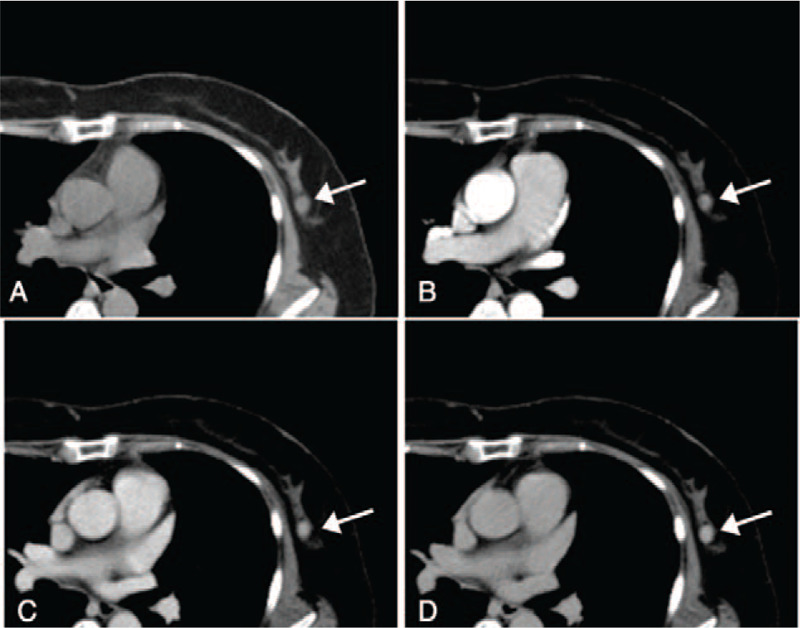
The dynamic CT image obtained 3 years before shows a pattern of gradual increase (arrows), which is known as the benign pattern. The mass presents as a homogeneous tumor with defined margin showing 30–33 Hounsfield Unit (HU) on non-contrast CT (a) and that increases to 87–92 HU in the arterial phase (b), 109–116 HU in the venous phase(c), and 119–123 HU in the capillary phase (d).

**Figure 3:**
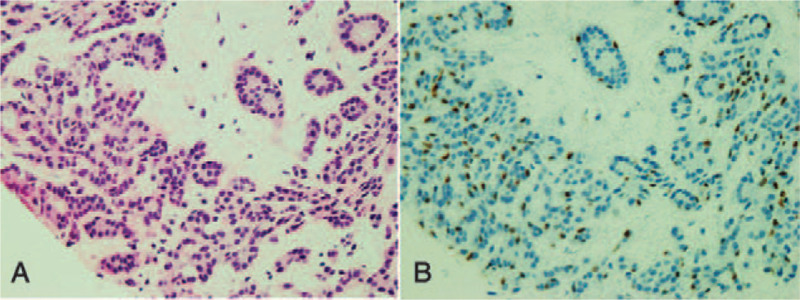
Pathologic examination from 3 years ago performed at the same time as the dynamic CT referred to in Fig. 2. A core needle biopsy shows fibroadenoma with a pericanalicular pattern. Epithelial ductal cells show proliferation and dissociation in myxoid stroma (a: H & E). Immunohistochemistry shows a reduction in myoepithelial cells (b: p63 stain).

Three years before this consultation, she had gone to another hospital to undergo another breast cancer screening program performed by the city (Table 1). Mammography at that time showed no remarkable change in the size or density of her left breast tumor. Dynamic CT imaging revealed a well-defined homogenous tumor that showed a pattern of gradual increase, which is known as the benign pattern (Fig. 2). A core needle biopsy performed that same day showed FA with a pericanalicular pattern but no pattern of malignancy even when assessed retrospectively. The specimen showed the proliferation and dissociation of small ductal epithelial cells (Fig. 3a) and a reduction in the number of myoepithelial cells on p63 immunohistochemical staining (Fig. 3b).

**Figure 4:**
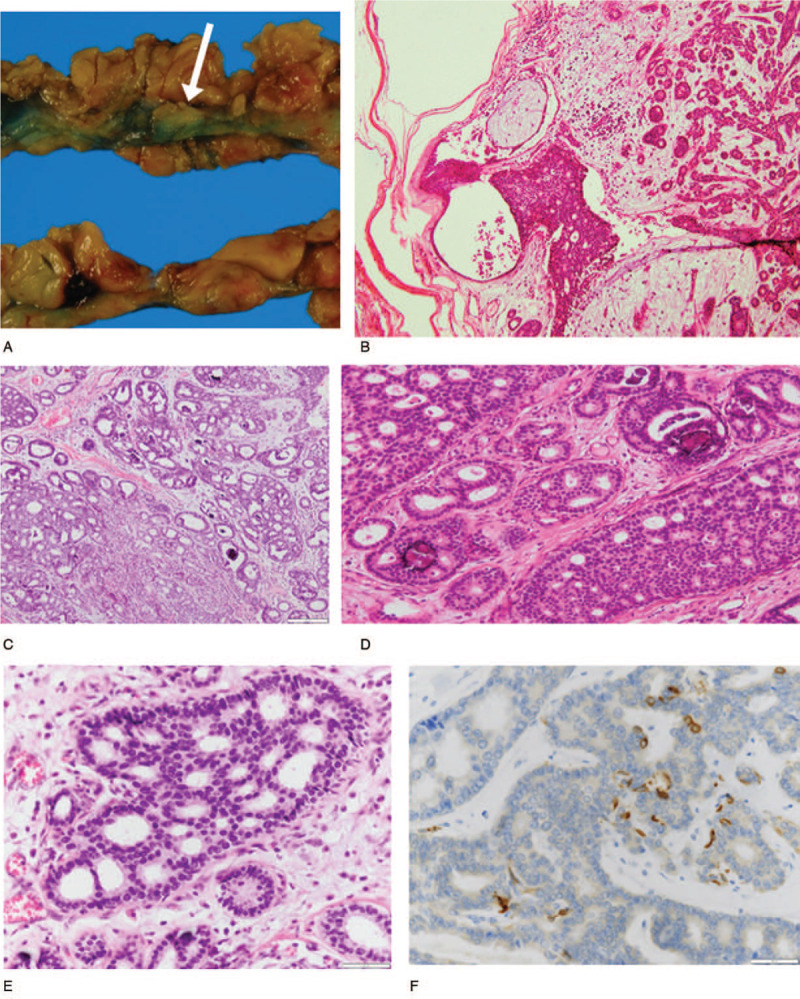
Macroscopic and microscopic findings of the present pathological examination. (a) The well-encapsulated ovoid tumor measuring 15 mm in diameter has a gray-white, slightly firm, and homogeneous cut surface (arrow). (b) The histopathological feature is that of a low-grade DCIS within a fibroadenomatous lesion. (c) The tumor is composed of malignant zones of dense proliferation of ductal epithelial cells showing cribriform, tubular, and solid growth patterns. (d) Typical cribriform pattern of the DCIS area with a few psammoma bodies. (e) High-power view of a cribriform area without mitotic figures. (f) Immunohistochemistry with CK5/6 stain shows extreme reduction of myoepithelial cells.

At the present consultation, mammography showed no remarkable change in the size or density of the tumor, although the secular change in background mammary tissue was reduced (Table 1). However, because the size of the tumor by US was slightly enlarged, we decided to perform MRI. Dynamic MRI showed a pattern of malignant enhancement, that is, a pattern of rapid enhancement and washout (Fig. 1f). The tumor showed high signal intensity on T2-weighted and diffusion-weighted images. So, we decided to perform a core needle biopsy, and the subsequent histopathological findings such as those of dense, epithelial proliferation with small ductal or cribriform growth pattern favored malignancy over its benign counterparts. The clinical stage was T1N0M0, Stage I. Then, breast-conserving therapy with sentinel lymph node biopsy of the left breast tumor was performed. The gross appearance of the excised specimen showed a well-encapsulated ovoid tumor measuring 15 mm in diameter with a gray-white, slightly firm, and homogenous cut surface (Fig. 4a). Histologically, a final diagnosis of low-grade DCIS within FA was obtained. The tumor encapsulated with thin fibrous tissue was composed of an intense proliferation of ductal cells showing cribriform, tubular, and solid growth patterns with fibroadenomatous myxoid stroma (Fig. 4b–f). The specimen's immune profile was estrogen receptor (ER), +(90%); PgR, −(3%); human epidermal receptor-2 (HER-2), −(membranous reaction, 0+); MIB-1 labeling index, 1% to 3%. Thus, the final specimen showed low-grade DCIS within a FA.

The patient underwent postoperative breast tangential irradiation therapy of 50 Gy for 5 weeks without any side effects and showed no signs of recurrence for 2 years.

## 3 Discussion

With an incidence ranging between 0.002% and 0.1%,^[1,2]^ carcinoma arising within a FA is very rare. Previous studies have reported that FA can evolve into different types of malignancy, such as lobular intraepithelial neoplasia, lobular carcinoma in situ, malignant phyllodes tumor, and microinvasive lobular carcinoma.^[3]^ Findings of malignancy inside a FA are usually those of carcinoma in situ: <15% are invasive breast cancers.^[4]^ Diaz et al reported that ductal and lobular types occurred with equal frequency in 105 cases surveyed.^[5]^ However, Fondo et al found that the majority of lesions were lobular carcinoma in situ (71%) and that 29% of the patients in their study had carcinoma of the contralateral breast.^[6]^ Wu et al reported the case of a 31-year-old patient with a diagnosis of DCIS arising from FA based on a core needle biopsy, and on tumor excision, invasive ductal carcinoma with lymph node metastasis was observed.^[4]^

Based on the clinicopathological presentation, FA can be divided into simple and complex types. Complex FA is characterized by cysts, sclerosing adenosis, epithelial calcification, or papillary apocrine change. Complex FA also increases the risk of breast cancer in women,^[3]^ with the relative risk reported to increase to 3.10.^[7]^ In general, FA is a long-term risk factor for breast cancer, and the risk increases in women with complex FA, proliferative disease, or a family history of breast cancer.^[8]^

Although Gollapalli et al reported that FAs containing foci of carcinoma in situ could be indistinguishable from benign lesions on imaging,^[3]^ dynamic MRI can detect the malignancy of a mammary tumor.^[7]^ However, the identification of malignant transformation from FA by other imaging modalities has not been discussed before to our knowledge. Here, new findings based on the present case show the importance of focusing on malignant change using diagnostic imagings even if the malignant changes occurring in FA are infrequent.

In the present case, US revealed slight tumor enlargement especially in thickness compared with the previous US examinations. The increase in thickness of the FA indicated malignant change because the tumor became harder due to rising cellular density, which was noted when the tumor was pressed on by the US probe. Usually, FA decreases in size year by year, especially in women in the post-menopausal state. Because US offers high-resolution imaging and is superior for measuring the exact size of a tumor in 3 dimensions, evaluation is easy when comparing images from previous studies. US is an inexpensive, radiation-free, and non-invasive modality, thus making it reasonable to perform frequent studies during the follow-up of FAs to determine whether malignant change is occurring.

In this case, mammography was not useful because the tumor remained almost equal in size over the 5 years, and no calcification was present on mammography.

In contrast, the pattern of dynamic CT/MRI enhancement changed from benign to malignant in only a 3-year interval. The pathological specimen confirmed to be FA transformed into a low-grade DCIS arising in a FA. The previous dynamic CT study showed a pattern of gradual increase known as the benign pattern. However, the time–intensity curve on the present dynamic MRI manifested a pattern of rapid enhancement and washout. This finding emphasizes the possibility that dynamic MRI studies can also be helpful in the identification of malignant changes occurring in a tumor. So, our case suggested that careful observation by US followed by the dynamic MRI is essential in the early diagnosis of the DCIS originating in the FA.

Pathologically, the fibroadenomatous findings of this case were characterized by intense proliferation and dissociation of small ductal epithelial cells and a reduction of myoepithelial cells in fibroadenomatous myxoid stroma. These findings can occur even in women of post-menopausal age and must be carefully followed-up because of the risk of malignant change.^[7]^

## Author contributions

**Conceptualization:** Hiroko Shojaku.

**Data curation:** Hiroko Shojaku.

**Formal analysis:** Hiroko Shojaku.

**Funding acquisition:** Hiroko Shojaku.

**Investigation:** Hiroko Shojaku, Ryota Hori, Toru Yoshida, Katsuo Shimada, Nobutatsu Takayanagi.

**Methodology:** Hiroko Shojaku, Ryota Hori, Toru Yoshida, Kazuhiro Matsui, Katsuo Shimada, Nobutatsu Takayanagi.

**Project administration:** Hiroko Shojaku.

**Resources:** Hiroko Shojaku.

**Software:** Hiroko Shojaku.

**Supervision:** Hiroko Shojaku, Kyo Noguchi.

**Validation:** Hiroko Shojaku, Kazuhiro Matsui, Nobutatsu Takayanagi.

**Visualization:** Hiroko Shojaku, Kazuhiro Matsui, Nobutatsu Takayanagi.

**Writing – original draft:** Hiroko Shojaku.

**Writing – review & editing:** Hiroko Shojaku.
